# Transcriptomic Analysis of the Highly Derived Radial Body Plan of a Sea Urchin

**DOI:** 10.1093/gbe/evu070

**Published:** 2014-04-02

**Authors:** Jennifer A. Wygoda, Yee Yang, Maria Byrne, Gregory A. Wray

**Affiliations:** ^1^Department of Biology, Duke University; ^2^School of Mathematics & Statistics, University of Sydney, Sydney, Australia; ^3^Schools of Medical & Biological Sciences, University of Sydney, Sydney, Australia

**Keywords:** RNA-seq, sea urchin, radial body plan, metamorphosis

## Abstract

With their complex life cycle and highly derived body plan, echinoderms are unique among bilaterians. Although early development has been intensively studied, the molecular mechanisms underlying development of the adult echinoderm and its unusual radial body plan are largely unknown. To investigate the evolution of developmental changes in gene expression underlying radial body plan development and metamorphosis, we assembled a reference transcriptome de novo and used RNA-seq to measure gene expression profiles across larval, metamorphic, and postmetamorphic life cycle phases in the sea urchin *Heliocidaris erythrogramma*. Our results present a high-resolution view of gene expression dynamics during the complex transition from pre- to postmetamorphic development and suggest that distinct sets of regulatory and effector proteins are used during different life history phases. These analyses provide an important foundation for more detailed analyses of the evolution of the radial adult body of echinoderms.

## Introduction

The body plan of echinoderms has radically diverged from other bilaterians: Although echinoderm larvae are bilaterally symmetric, juveniles and adults are characterized by a pentaradial body plan. How this unique radial morphology relates to the body plan of other bilaterians, particularly hemichordates and chordates, has long been a debate among evolutionary biologists (Hyman 1955; Hotchkiss 1998; Morris 1999; Peterson et al. 2000; Smith 2008). Although the molecular and cellular mechanisms underlying larval development in echinoderms have been well studied, particularly in the model sea urchin *Strongylocentrotus purpuratus*, almost nothing is known about the molecular basis of adult body plan development or metamorphosis in these animals. This is due in large part to the complex life cycle of echinoderms and the difficulty of maintaining larvae through metamorphosis, which can take several weeks or months involving intensive rearing and feeding (Wray et al. 2004). These limitations make investigations of adult body plan development and metamorphosis intractable for most echinoderms.

The sea urchin *Heliocidaris erythrogramma* employs an alternative developmental strategy in which the feeding larval stage is omitted and adult development is greatly accelerated (Williams and Anderson 1975; Raff 1992). Although embryonic and larval stages have been substantially modified in this species, many aspects of development of the adult radial body plan remain conserved between *H. erythrogramma* and related sea urchins with fully indirect development (Williams and Anderson 1975; Ferkowicz and Raff 2001; Smith et al. 2009). *Heliocidaris erythrogramma* is an ideal species to investigate adult body plan development and metamorphosis in echinoderms because of its rapid development to the pentameral body plan within a week and amenability to experimental perturbation (Zhou et al. 2003; Wilson et al. 2005; Smith et al. 2008). Its derived life history strategy of nonfeeding development has also been the focus of many studies of alternative developmental modes in echinoderms (Wray and Raff 1990, 1991; Raff 1992; Byrne et al. 1999; Smith et al. 2007; Love et al. 2008). Thus, the availability of genomic resources for *H. erythrogramma* will advance investigations of body plan evolution and metamorphosis, as well as studies of life history evolution in marine invertebrates.

Here, we present a reference transcriptome for *H. erythrogramma* that was assembled de novo from a broad range of developmental stages. We used RNA-seq to measure transcription profiles across a developmental time-course in order to explore gene expression changes related to adult body plan development and metamorphosis. Previous studies have provided insight into the evolution of the radial body plan of echinoderms from bilateral ancestors based on the development of the adult nervous system in sea urchins (Raff and Popodi 1996; Morris 1999; Sly et al. 2002; Nielsen et al. 2003). Therefore we focused in particular on the expression of neurogenesis genes, as organization of the central nervous system closely parallels that of the body plan across animal groups (reviewed by Holland 2003; Angerer et al. 2011). We were also interested in addressing the question: Are larval neurogenesis genes repurposed during development of the adult nervous system or is there a suite of adult-specific neurogenesis transcription factors? Our analyses highlight a dramatic shift in gene expression dynamics during the transition from pre- to postmetamorphic development in *H. erythrogramma* and provide an important framework for future work investigating evolutionary changes the functional role of specific genes in radial body plan development.

## Results

### A Highly Dynamic Developmental Transcriptome

**Fig. 1.—:**
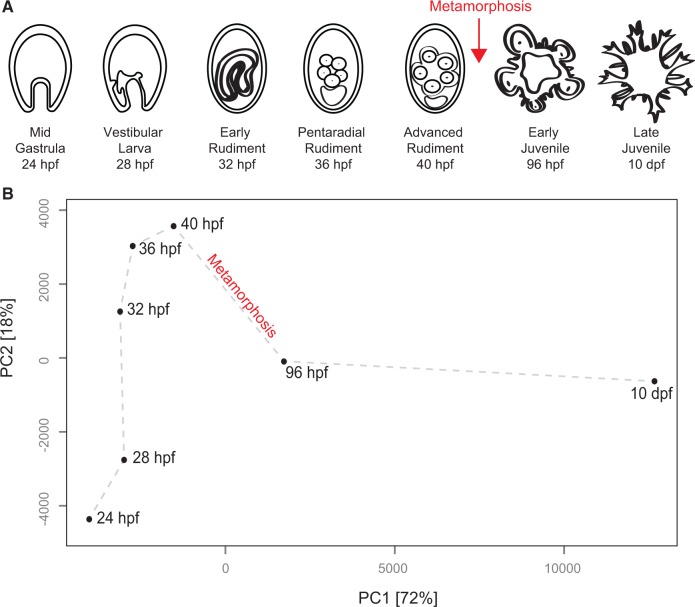
Developmental transcriptome in *Heliocidaris erythrogramma*. (*A*) Our developmental time-course included seven stages spanning premetamorphic (24, 28, 32, 36, and 40 hpf) and postmetamorphic development (96 hpf and 10 dpf). (*B*) PC analysis of gene expression averaged across biological replicates. PC1 explains 72% of the overall variation and clearly separates pre- from postmetamorphic stages, whereas PC2 explains 18% of the variation. The gray line is superimposed to show that overall variation in gene expression between stages recapitulates our developmental time-course.

Our transcriptome time-course in *H. erythrogramma* included seven developmental stages spanning larval, metamorphic, and postmetamorphic life cycle phases (fig. 1*A*). Development of the adult body plan begins by 28 hours past fertilization (hpf), pentaradial symmetry is readily apparent by 36 hpf, and metamorphosis takes place by 96 hpf.

To assess the global variation in gene expression during development, we performed a principal components (PC) analysis (fig. 1*B*). The first PC explained 72% of the variation in gene expression data between time points. Strikingly, most of the variation explained by PC1 distinguished postmetamorphic stages. In contrast, PC2 explained nearly all of the variance among premetamorphic stages. These results suggest that overall variation in gene expression between stages recapitulates morphological development and that more changes take place during metamorphosis than at other times during radial body plan development. Therefore, we focused attention on changes that occur during the transition from pre- to postmetamorphic development (i.e., from the advanced rudiment to early juvenile stage), as well as changes that take place between the early and late juvenile stages.

**Fig. 2.—:**
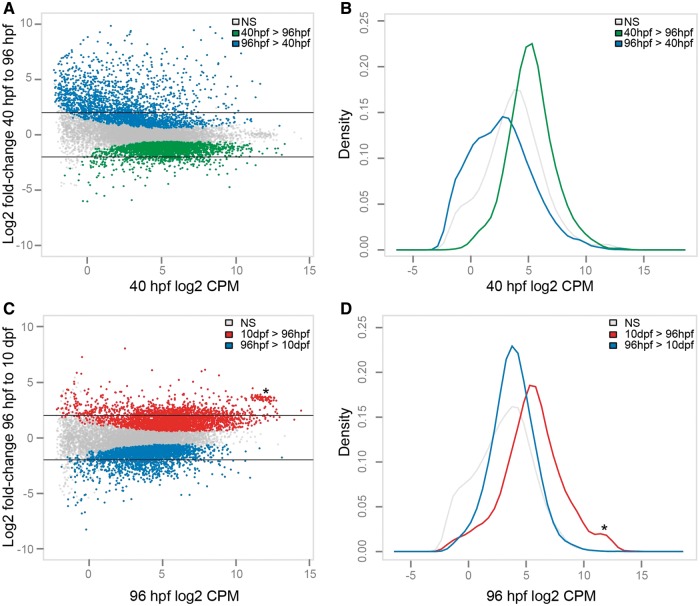
Metamorphic and postmetamorphic life cycle stages are characterized by dynamic gene expression patterns. (*A*) MA plot showing the average expression level (in counts per million) of all 13,017 genes at 40 hpf and the log_2_ fold-change of each gene from 40 to 96 hpf. Genes with a significant decrease in expression at an FDR of 5% are green, whereas genes with a significant increase in expression are blue. Nonsignificant genes are in gray. Horizontal black bars indicate genes that exhibited at least a 4-fold change in expression (log_2_FC = 2). (*B*) Kernel density curves of the expression of genes that significantly decreased (green), increased (blue) or did not change (gray) from 40 to 96 hpf at an FDR of 5%. (*C*) MA plot showing the average expression level at 96 hpf and the log_2_FC of each gene from 96 to 10 dpf. Genes with a significant decrease in expression at an FDR of 5% are blue, whereas genes with a significant increase in expression are red. Nonsignificant genes are in gray. (*D*) Kernel density curves of the expression of genes that significantly decreased (blue), increased (red) or did not change (gray) from 96 hpf to 10 dpf at an FDR of 5%.

Metamorphosis takes place between the advanced rudiment and early juvenile stages. Across this transition, 4,544 genes (33.5% of those examined) were differentially expressed after controlling for a false discovery rate (FDR) of 5% (fig. 2*A*). Of these, 2,322 genes were downregulated during metamorphosis and 2,222 genes were upregulated. From the skewed distribution of these upregulated genes (fig. 2*B*, blue kernel), it is evident that a large fraction of genes with low-to-moderate expression prior to metamorphosis were upregulated in the early juvenile. This provides further evidence that a dramatic shift in overall expression profile occurs during metamorphosis.

We also observed many significant changes in gene expression during postmetamorphic development: 2,679 genes were downregulated between early juveniles and late juveniles, whereas 2,960 genes were upregulated after controlling for an FDR of 5% (fig. 2*C*). Postmetamorphic distributions of down- and upregulated genes were similar though a large number of highly expressed genes in the early juvenile tended to further increase in the late juvenile (fig. 2*D*). A striking example of this trend is a conspicuous cluster of 63 genes (fig. 2*C* and *D*, asterisk). All but two of the genes in this cluster encode ribosomal proteins: *Sm37* and *Chrna9* encode a spicule matrix protein and a cholinergic receptor, respectively. Because ribosomal proteins are essential to protein synthesis, a large increase in their expression during postmetamorphic development is consistent with the increased demands of growth and metabolism in late juveniles.

### Extensive Changes in Functional Categories Accompany the Pre- to Postmetamorphic Transition

Given the marked differences in gene expression between pre- and postmetamorphic development, we next asked whether certain biological processes were enriched among upregulated genes in our comparisons of advanced rudiment, early juvenile, and late juvenile stages. We performed a categorical enrichment analysis using a hypergeometric test and the PANTHER ontology database (Thomas et al. 2003) to test whether the top 10% of differentially expressed genes in each comparison was enriched for particular biological process categories. Our results show a clear distinction in enriched categories between stages.

First, among genes that were expressed at higher levels in the advanced rudiment stage, we found enriched categories related to RNA and DNA metabolism, RNA processing, and cell division (supplementary table S1, Supplementary Material online). In contrast, genes that increased expression in early juveniles were significantly enriched for categories related to cell signaling, development, neurogenesis, transport, and reproduction (supplementary table S2, Supplementary Material online). This difference likely reflects the use of distinct sets of regulatory and effector proteins in pre- and postmetamorphic development.

Next, we compared categorical enrichments of upregulated genes between early and late juveniles. We found 31 significantly enriched categories of genes expressed at higher levels in early juveniles (supplementary table S3, Supplementary Material online), several of which were represented in our earlier analysis comparing this stage with the advanced rudiment stage (e.g., nervous system development and reproduction). Categories uniquely enriched relative to late juveniles were related to morphogenesis and protein modification. Conversely, we found 13 enriched categories in late juveniles (supplementary table S4, Supplementary Material online). A number of these were related to metabolic processes and the generation of precursor metabolites and energy, again indicating that late juveniles experience increased requirements of growth and metabolism.

**Fig. 3.—:**
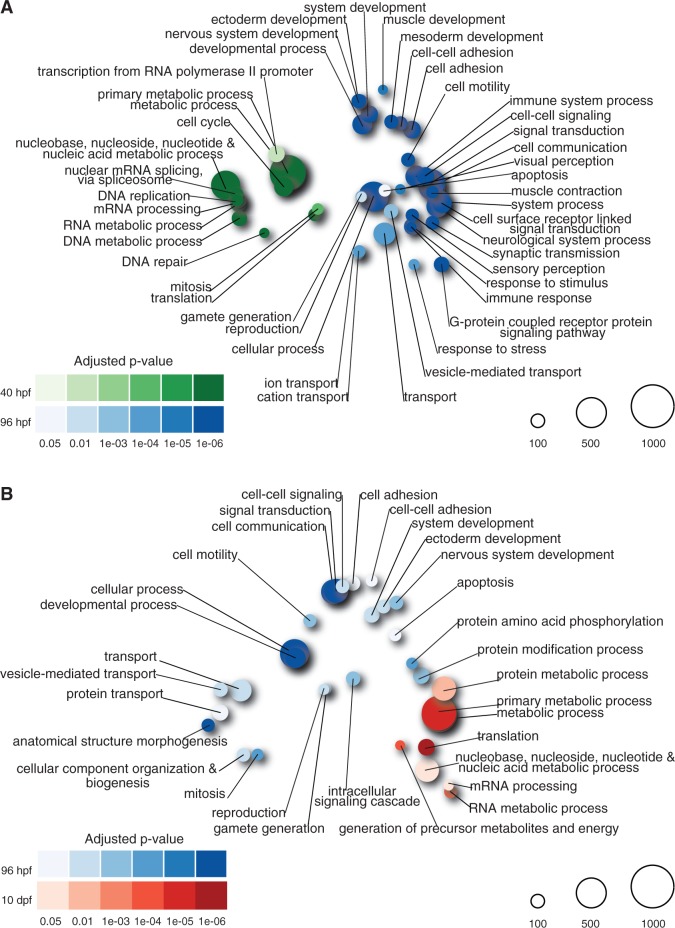
Advanced rudiment, early juvenile, and late juveniles exhibit stage-specific enrichment of PANTHER biological process categories. (*A*) Bubble plot showing significantly enriched PANTHER biological process categories at 40 hpf (green) compared with 96 hpf (blue) at an FDR of 5%. Significant categories were identified by a hypergeometric test for the top 10% of a probability distribution. For each category, significance is represented by bubble color and occupancy by bubble size. For clarity, only categories with at least 50 members are shown (see supplementary tables S1 and S2, Supplementary Material online, for additional categories). (*B*) Bubble plot showing significantly enriched categories at 96 hpf (blue) compared with 10 dpf (red) at an FDR of 5% (see supplementary tables S3 and S4, Supplementary Material online, for additional categories).

In order to visualize the hierarchical and overlapping nature of enriched categories between pre- and postmetamorphic stages, we generated a distance matrix of PANTHER biological process categories (see Materials and Methods). We then used multidimensional scaling to display the distance matrix for significantly enriched categories as a “bubble plot.” For each category, significance is represented by bubble color and occupancy by bubble size. This visualization method allowed us to identify an interesting trend: Although we observed a clear delineation between significantly enriched categories in the advanced rudiment stage compared with early juveniles (fig. 3*A*), the separation between enriched categories was less defined between the early and late juvenile stages (fig. 3*B*). This observation is consistent with the results of the PC analysis (fig. 1).

### Broad Patterns of Gene Expression Changes during Development

**Fig. 4.—:**
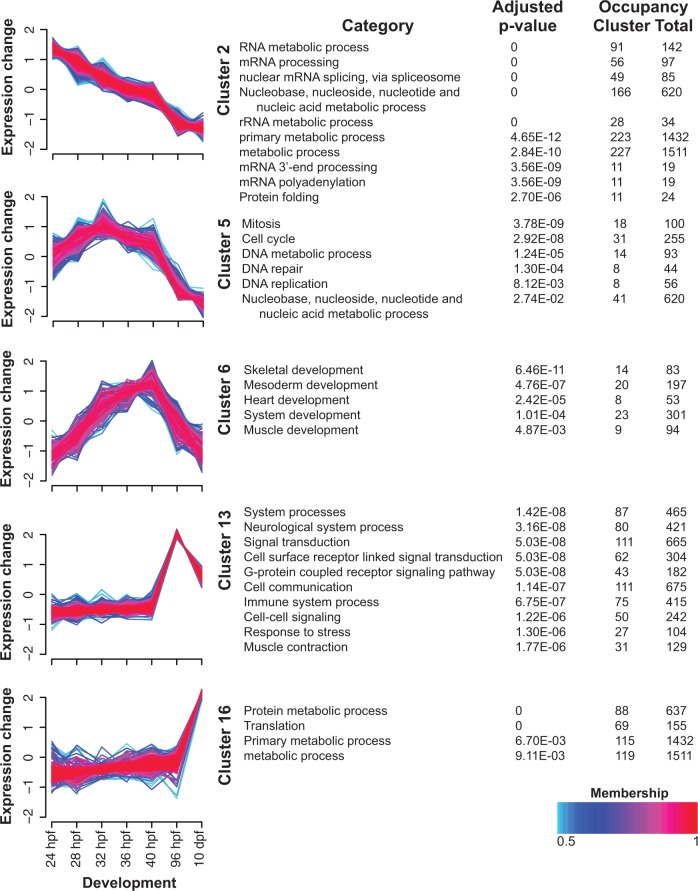
Fuzzy c-means clustering identifies general patterns of gene expression across development. To illustrate major patterns of gene expression, five clusters detected by fuzzy c-means clustering of the average expression value of 4,421 genes (log_2_FC ≥ 2, FDR ≤ 0.001) are shown (see supplementary fig. S1, Supplementary Material online, for additional clusters). The ms of a given gene within a cluster is represented by color, with red (ms = 1) indicating high association. For each cluster, significantly enriched PANTHER biological process categories identified by a Wilcoxon signed rank test are shown. For clarity, only the top ten categories are displayed (see supplementary table S5, Supplementary Material online, for additional categories).

Although our comparisons of advanced rudiment, early juvenile, and late juvenile stages provide important insights into the differences in pre- and postmetamorphic development, they do not take the full time-course into account. To further investigate the large-scale patterns of gene expression across juvenile body plan development and metamorphosis, we used fuzzy c-means clustering to group differentially expressed genes (log fold-change ≥ 2, FDR ≤ 0.001) into 16 distinct clusters (see Materials and Methods). We kept 3,316 clustered genes for further analysis based on their membership score (ms) within a cluster (ms ≥ 0.5). The majority of clusters exhibited both stage-specific expression profiles (supplementary fig. S1, Supplementary Material online) and significant enrichment of PANTHER biological process categories (Wilcoxon rank test; supplementary table S5, Supplementary Material online). Generally, the patterns that we observed could be summarized by Clusters 2, 5, 6, 13, and 16 (fig. 4).

Following gastrulation, genes related to RNA processing, metabolic processes, and translation decrease steadily throughout development (Cluster 2). Subsequently, genes related to cell cycle and DNA replication generally increase in expression (Cluster 5), followed by genes related to various developmental processes just prior to metamorphosis (Cluster 6). It is important to note, however, that although many of the genes associated with these enriched developmental categories play known roles in sea urchin development, they are not necessarily involved in the specific functions indicated by the category name as these associations are based on developmental studies in model organisms. The full list of associations between PANTHER biological process categories and each gene annotated in this study is provided as a supplementary table S6, Supplementary Material online (see Materials and Methods).

Immediately following metamorphosis, we observed an enrichment of neural, immune, signaling, and development categories (Cluster 13). Cluster 16 includes genes that remain quiescent throughout most of development and peak in expression in the late juvenile. Interestingly, many of these genes are related to metabolic processes and translation, categories that were also enriched in Cluster 2. Together, these results indicate that stage-specific shifts in gene expression may reflect specific functional demands of individual developmental processes as we observed coordinated changes in enriched functional classes between clusters. Given that almost 33% of clustered genes were unclassified, however, much remains to be learned about the genes expressed during adult plan development and metamorphosis.

These results also reveal that genes involved in developmental processes are primarily expressed in three broad phases. First, several known regulators of larval development in sea urchins (e.g., *chordin*, *hes*, *even-skipped*, *lefty*, *foxY*) declined throughout our time-course (Cluster 1, supplementary fig. S1, Supplementary Material online). A second set of genes involved in skeletal and mesoderm development (e.g., *delta*, *hox8*, *msp130*, *myc*, *ets1/2*) rise in expression through development of the radial juvenile and peak prior to metamorphosis (fig. 4, Cluster 6). Finally, the third set of developmental genes, the majority of which are involved in neural development (e.g., *vacht*, *lmx1*, *pax1/9*, *hb9*, *lhx3-4*), is activated following metamorphosis in the early juvenile (fig. 4, Cluster 13).

### Neurogenesis Transcription Factors Have Distinct Phases of Expression

Given the close relationship between body plan and nervous system anatomy (Nielsen 1999; Minsuk and Raff 2002; Lowe 2008), we next examined the expression of neurogenesis transcription factors during our time-course. Specifically, we were interested in addressing the question: Are larval neurogenesis genes repurposed during development of the adult nervous system or is there a suite of adult-specific neurogenesis transcription factors?

**Fig. 5.—:**
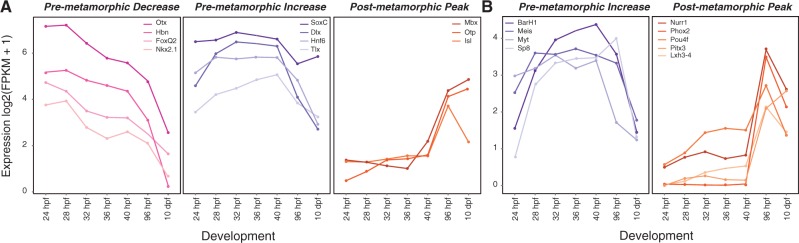
Expression of neural transcription factors during juvenile development. (*A*) Several larval neurogenesis genes are also expressed during juvenile development and generally exhibit a premetamorphic decrease, premetamorphic increase, or a postmetamorphic peak of expression. Each line represents the average expression value (in fragments per kilobase of transcript per million mapped reads) for an individual gene. (*B*) Juvenile-specific neurogenesis transcription factors exhibit either a premetamorphic increase or a postmetamorphic peak of expression.

First, we analyzed the expression of 35 genes known to encode transcription factors expressed during development of the larval nervous system (Burke et al. 2006) and found that all are also expressed in postmetamorphic stages (supplementary table S7, Supplementary Material online). This suggests that larval neurogenesis genes are also deployed during development of the adult nervous system. We were particularly interested in identifying examples of premetamorphic decrease, premetamorphic increase, and postmetamorphic peaks of expression (examples are highlighted in fig. 5*A*). Genes that gradually declined following gastrulation (e.g., *orthodenticle*, *homeobrain*, *foxQ2*, and *nkx2.1*) likely have a limited or early role in adult neurogenesis. However, it is also possible that the spatial expression domains of these genes have decreased as particular neural tissues become more defined during development. We also identified a handful of genes that increased in expression during rudiment development (e.g., *soxC*, *distal-less*, *hnf6*, and *tail-less*) and others that peaked in expression after metamorphosis (e.g., *mbx*, *orthopedia*, and *islet*).

Several genes were previously identified in the *S. purpuratus* genome as putative neurogenesis transcription factors, but lack expression in the larval nervous system (Burke et al. 2006). Therefore, we hypothesized that they may be involved in development of the adult nervous system. We examined the expression of 18 of these putative neurogenesis genes across our time-course (supplementary table S7, Supplementary Material online). A few of these transcription factors exhibited a premetamorphic peak in expression during rudiment development (e.g., *barh1*, *meis*, *myt*, and *sp8*) (fig. 5*B*). However, the majority either peaked in expression after metamorphosis (e.g., *nurr1*, *phox2*, *pou4f*, *pitx3*, and *lxh3-4*) or were expressed at a low level throughout our time-course (e.g., *neuroD* and *engrailed*).

These results suggest that larval neurogenesis genes may be involved in adult nervous system development, along with an adult-specific set of neurogenesis transcription factors. However, future work is necessary to identity the spatial expression patterns of these transcription factors in the developing adult body plan and to determine whether these genes do in fact have a role in adult neurogenesis. Investigations of the functional importance of these genes for different stages of adult neural development will be critical to our understanding of the echinoderm nervous system and how it has evolved for this highly derived body plan (Morris 1999; Nielsen et al. 2003).

## Conclusions and Future Directions

To investigate developmental changes in gene expression related to radial body plan development and metamorphosis in the sea urchin *H. erythrogramma*, we analyzed the transcriptome across larval, metamorphic, and postmetamorphic life cycle phases. The results suggest that the most extensive changes in transcript abundance occur during and after metamorphosis, indicating that these phases of development are more complex than earlier ones. Interestingly, our PC analysis indicates that larval stages were overall more similar to each other in transcript composition than any is to postmetamorphic stages. Together with the categorical enrichment analyses, this may reflect the use of somewhat distinct sets of regulatory and effector proteins in pre- and postmetamorphic development. Nonetheless, the separation of gene sets is far from absolute. For instance, although sea urchin larvae and adults develop nervous systems independently (Chia and Burke 1978), several genes encoding key regulators of larval nervous system development are also expressed during and after metamorphosis. It also seems likely that some of the global shifts in gene expression across stages reflect changes in broad functional demands of developmental processes as we observed coordinated changes in particular functional classes of genes.

Our results provide a foundation for future studies to carry out more detailed analyses of the evolution of the radial adult body of echinoderms. Although echinoderms clearly evolved from bilaterian ancestors, their complex life cycle and highly derived adult morphology make it difficult to reconstruct the origins of this unusual radial body plan (Smith 2008). The expression profiles for genes that pattern body axes and the central nervous system can provide powerful insights into the evolution of enigmatic body plans as work with hemichordates has so elegantly demonstrated (Lemons et al. 2010; Pani et al. 2012). The evolution of life history modes is another area of interest for future studies. The shift from feeding to nonfeeding development in the genus *Heliocidaris* is now one of the most thoroughly studied cases of this kind in terms of molecular and developmental mechanisms (Wray and Raff 1990; Raff 1992; Byrne et al. 1999; Wilson et al. 2005; Smith et al. 2007, 2008, 2009). Comparing transcriptomes between species that employ different developmental modes has the potential to highlight some of the shifts in gene expression that underlie ecologically relevant evolutionary changes in development.

## Materials and Methods

### Sample Collection

*Heliocidaris erythrogramma* adults were collected near Sydney, Australia and larvae were reared following standard protocols. We sampled seven developmental stages, replicated in triplicate, for this study: Gastrula (24 hpf), vestibular larva (28 hpf), early rudiment (32 hpf), pentaradial rudiment (36 hpf), advanced rudiment (40 hpf), early juvenile (96 hpf), and late juvenile (10 days past fertilization [dpf]). For each stage, approximately 300 larvae were placed in RNAlater (Qiagen) and shipped to Duke University for RNA preparation and sequencing.

### RNA Preparation and Sequencing

RNA was extracted for each sample using an RNeasy kit (Qiagen). RNA quantity was measured using a NanoDrop and quality was assessed with a BioAnalyzer. Four micrograms of total RNA was used as input for the Illumina Tru-Seq Library Preparation Kit and libraries were prepared according to the manufacturer’s instructions. Libraries were sequenced on an Illumina Hi-Seq 2000 at the Duke Genome Sequencing and Analysis Shared Resource. We sequenced two of the three replicates with 50-pb paired-end sequencing on two lanes (approximately 416 million reads per lane) and the third replicate with 50-bp single-end sequencing on one lane (approximately 223 million reads per lane).

### Transcriptome Assembly and Annotation

We used Trinity to carry out de novo assembly of a reference transcriptome from the paired-end reads (N50 = 1,769, minimum contig length = 201, maximum contig length = 9,873). Putative open reading frames were predicted with OrfPredictor (Min et al. 2005). Peptide sequences obtained from OrfPredictor were reciprocally best blasted (RBB) against the *S. purpuratus* peptide database (version 3.1) with an *e*-cutoff of 1 e-10. Because de novo assemblies often include fragmented contigs, contigs not identified by RBB were blasted separately against the database. We were able to annotate 13,548 unique genes in our assembly. Of these, we were able to assign 8,469 genes to biological process categories downloaded from the PANTHER gene ontology database version 8.1 based on curated associations of these categories with *S. purpuratus* gene models (supplementary table S6, Supplementary Material online) (Thomas et al. 2003).

### Read Mapping, Data Normalization, and Differential Expression

Quality-filtered RNA-seq reads (left-reads/single-reads) were mapped to the reference transcriptome assembly with Bowtie (Langmead et al. 2009) and abundance estimation was performed with RSEM (Li and Dewey 2011). For contigs that RBB as the same gene, we combined counts and computed length-weighted FPKM (fragments per kilobase of transcript per million mapped reads). The calcNormFactors function of the R package edgeR (Robinson et al. 2009) was used to normalize gene counts for the remaining 13,017 genes and evaluate stage-specific differential expression. The edgeR functions estimateCommonDisp and estimateTagwiseDisp were used to estimate dispersion. Gene models with fewer than 5 counts were excluded from this analysis and FDRs were controlled using the Benjamini–Hochberg method (Benjamini and Hochberg 1995) at an FDR of 5%.

### Categorical Enrichment and Cluster Analyses

The “hypergeometric.py” script of the python package “pyEnrichment” (https://github.com/ofedrigo/pyEnrichment, last accessed December 2014) was used to test for significant enrichments of the top 10% of differentially expressed genes between advanced larvae, early juveniles, and late juveniles. This threshold was necessary given the large number of differentially expressed genes between developmental stages. To visualize categorical enrichments, we used multidimensional scaling to calculate an optimal 2D arrangement of categories based on a distance matrix of between-category semantic scores using “makeDendrogram.py” (pyEnrichment) and scripts provided by Daniel Runcie (personal communication). For each category, significance is represented by bubble color and occupancy by bubble size.

For cluster analysis, mean FPKM values were clustered using the R package Mfuzz (Kumar and Futschik 2007), which performs fuzzy c-means clustering. Only genes with significant differences in expression between at least two time points (log_2_FC ≥ 2, FDR ≤ 0.001) were used as input for the clustering (4,421 genes total). The number of clusters was set to 16 and the fuzzifier coefficient set to 1.55. The optimal number of clusters was determined by first plotting the minimum centroid distance across a range of cluster values and then observing the cluster value at which this distance plateaued (supplementary fig. S2, Supplementary Material online). Following cluster analysis, genes with an ms of at least 0.5 were plotted and used as input for categorical enrichment analysis (3,316 genes total, including 1,102 genes of unknown function). We used a Wilcoxon signed rank test implemented with the “wilcoxon.py” script of the pyEnrichment package to test whether particular biological process categories were enriched within a given cluster.

## Supplementary Material

Supplementary tables S1–S7 and figures S1 and S2 are available at *Genome Biology and Evolution* online (http://www.gbe.oxfordjournals.org/).

Supplementary Data
