# Supplementary material for: Transcriptomic Analysis of the Highly Derived Radial Body Plan of a Sea Urchin
Source: Genome Biol Evol. 2014 Apr 2;6(4):964–73. doi: 10.1093/gbe/evu070 (PMC4007537; doi:10.1093/gbe/evu070)
Supplement: Supplementary Data [file supp_6_4_964__index.html]

Transcriptomic Analysis of the Highly Derived Radial Body Plan of a Sea Urchin — Supplementary Data 

# Transcriptomic Analysis of the Highly Derived Radial Body Plan of a Sea Urchin

## Supplementary Data

files

**Files in this Data Supplement:**

- Supplementary Data - pdf file
- Supplementary Data - pdf file
- Supplementary Data - xlsx file
- Supplementary Data - xlsx file
- Supplementary Data - xlsx file
- Supplementary Data - xlsx file
- Supplementary Data - xlsx file
- Supplementary Data - xls file
- Supplementary Data - xlsx file
