# Supplementary figures and images for: Transcriptomic Analysis of the Highly Derived Radial Body Plan of a Sea Urchin
Source: Genome Biol Evol. 2014 Apr 2;6(4):964–73. doi: 10.1093/gbe/evu070 (PMC4007537; doi:10.1093/gbe/evu070)

FIGURE S1

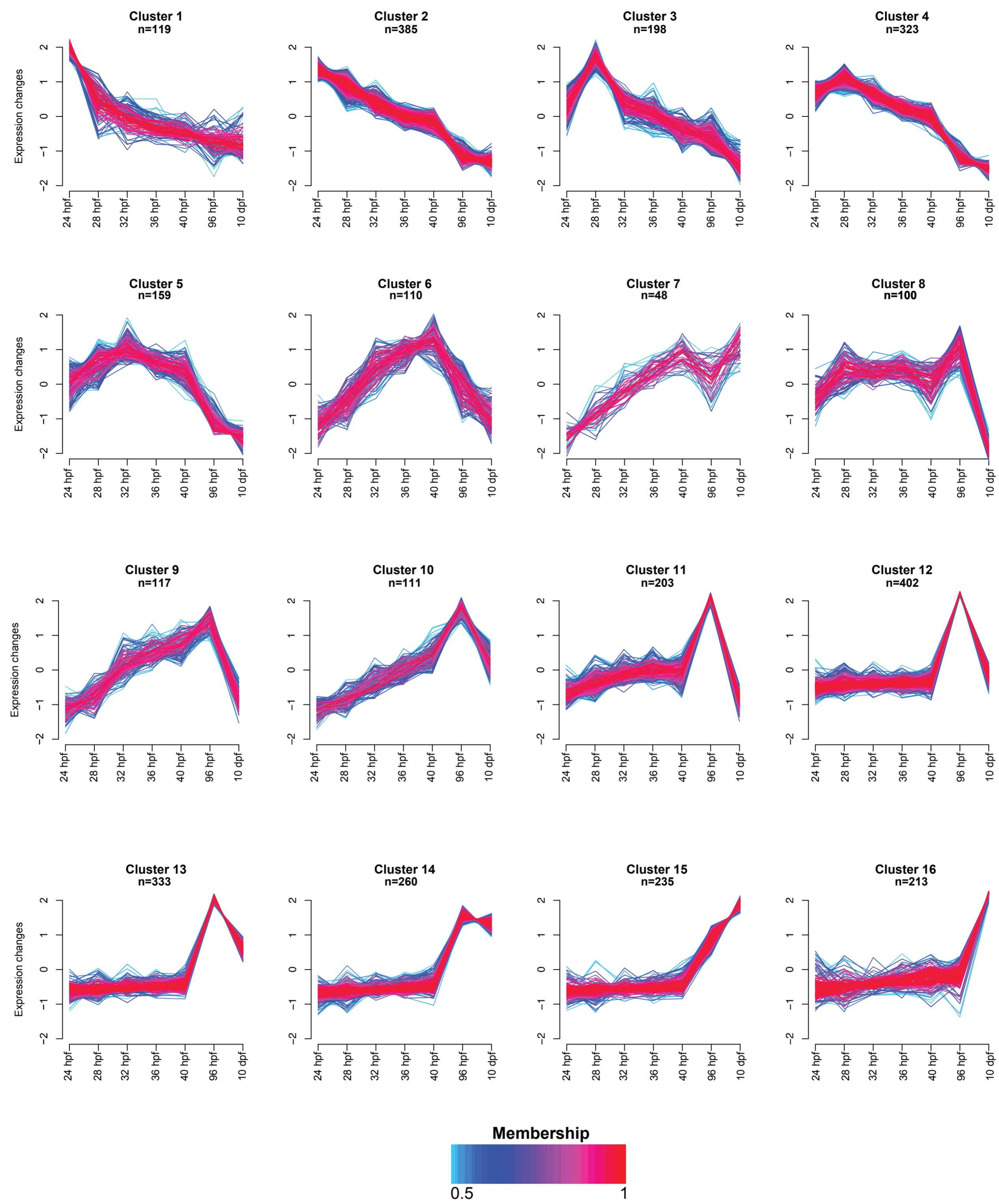

Supplement: Supplementary Data [file supp_evu070_FigureS1.pdf]

FIGURE S2

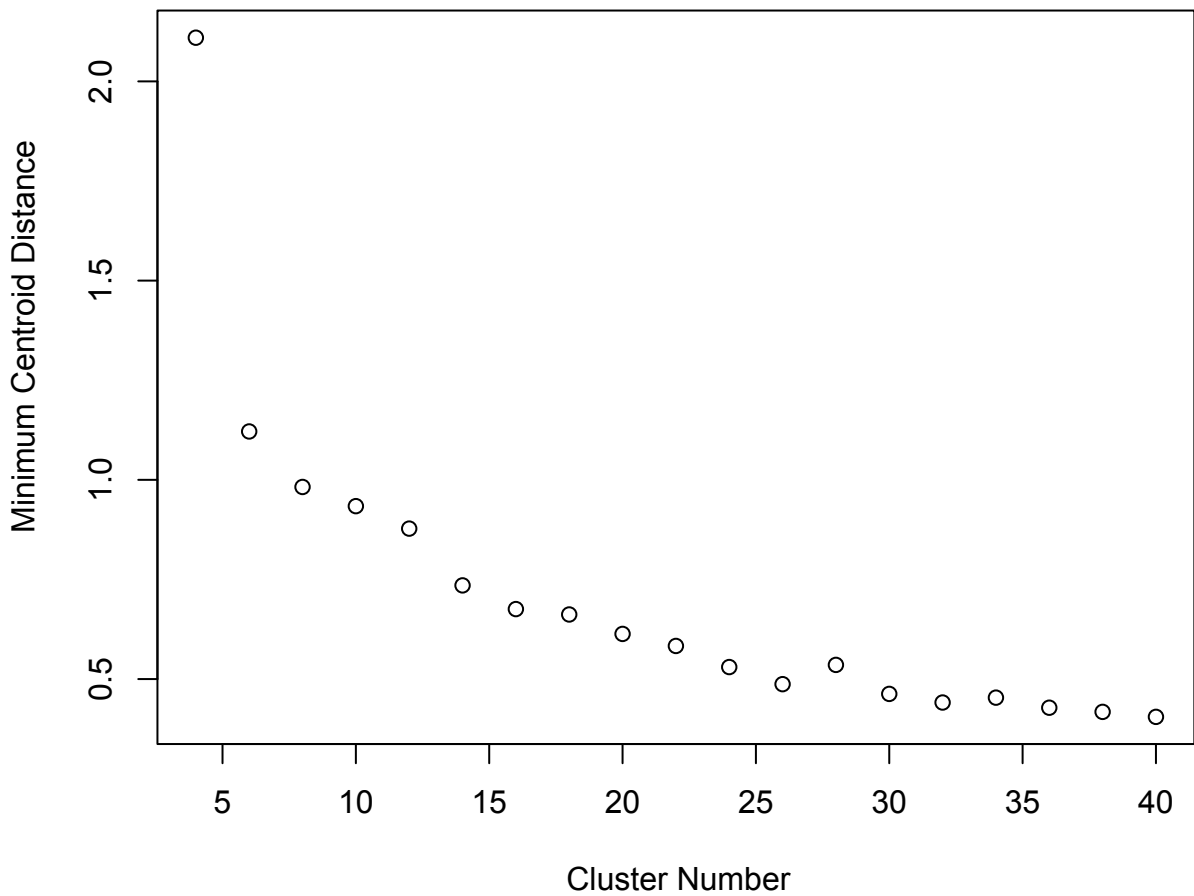

Supplement: Supplementary Data [file supp_evu070_FigureS2.pdf]
